# *catena*-Poly[[tris­(4-fluoro­benz­yl)tin(IV)]-μ-2-[(piperidin-1-yl)carbothio­ylsulfan­yl]acetato-κ^2^ *O*:*O*′]

**DOI:** 10.1107/S1600536810028321

**Published:** 2010-07-21

**Authors:** Chun Thy Keng, Kong Mun Lo, Seik Weng Ng

**Affiliations:** a Department of Chemistry, University of Malaya, 50603 Kuala Lumpur, Malaysia

## Abstract

Adjacent units of the title polymeric complex, [Sn(C_7_H_6_F)_3_(C_8_H_12_NO_2_S_2_)], are bridged by the carboxyl­ate ion into a helical chain running along the *b* axis. The Sn(IV) atom shows a distorted *trans*-C_3_SnO_2_ trigonal-bipyramidal coordination and is displaced by 0.113 (2) Å out of the C_3_Sn girdle in the direction of the covalently bonded O atom. The ring is disordered of two positions with an occupancy of 0.631 (4) for the major occupied site.

## Related literature

For a comment on the repeat distance of carboxyl­ate-bridged trialkyl­tin carboxyl­ates, see: Ng *et al.* (1989 ▶).
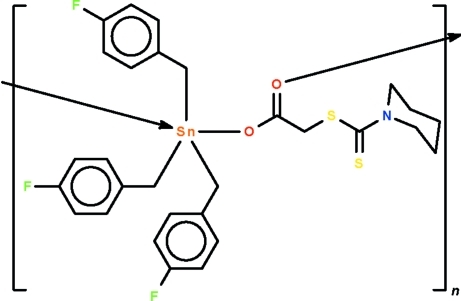


## Experimental

### 

#### Crystal data

[Sn(C_7_H_6_F)_3_(C_8_H_12_NO_2_S_2_)]
*M* *_r_* = 664.35
Monoclinic, 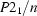

*a* = 11.8016 (7) Å
*b* = 10.4572 (6) Å
*c* = 23.0334 (13) Å
β = 94.013 (1)°
*V* = 2835.6 (3) Å^3^
*Z* = 4
Mo *K*α radiation
μ = 1.10 mm^−1^
*T* = 100 K
0.30 × 0.25 × 0.20 mm

#### Data collection

Bruker SMART APEX diffractometer
Absorption correction: multi-scan (*SADABS*; Sheldrick, 1996 ▶) *T*_min_ = 0.735, *T*_max_ = 0.811
25437 measured reflections
6498 independent reflections
5328 reflections with *I* > 2σ(*I*)
*R*_int_ = 0.038

#### Refinement

*R*[*F* ^2^ > 2σ(*F* ^2^)] = 0.034
*wR*(*F* ^2^) = 0.077
*S* = 1.05
6498 reflections
371 parameters
17 restraints
H-atom parameters constrained
Δρ_max_ = 0.50 e Å^−3^
Δρ_min_ = −1.19 e Å^−3^

### 

Data collection: *APEX2* (Bruker, 2009 ▶); cell refinement: *SAINT* (Bruker, 2009 ▶); data reduction: *SAINT*; program(s) used to solve structure: *SHELXS97* (Sheldrick, 2008 ▶); program(s) used to refine structure: *SHELXL97* (Sheldrick, 2008 ▶); molecular graphics: *X-SEED* (Barbour, 2001 ▶); software used to prepare material for publication: *publCIF* (Westrip, 2010 ▶).

## Supplementary Material

Crystal structure: contains datablocks global, I. DOI: 10.1107/S1600536810028321/bt5300sup1.cif

Structure factors: contains datablocks I. DOI: 10.1107/S1600536810028321/bt5300Isup2.hkl

Additional supplementary materials:  crystallographic information; 3D view; checkCIF report

## Figures and Tables

**Table:** 

Sn1—C1	2.144 (3)
Sn1—C8	2.155 (3)
Sn1—C15	2.158 (3)
Sn1—O1	2.175 (2)
Sn1—O2^i^	2.339 (2)

**Table:** 

O1—Sn1—O2^i^	170.59 (7)

Symmetry code: (i) 
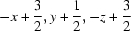
.

**Fig. 1.**
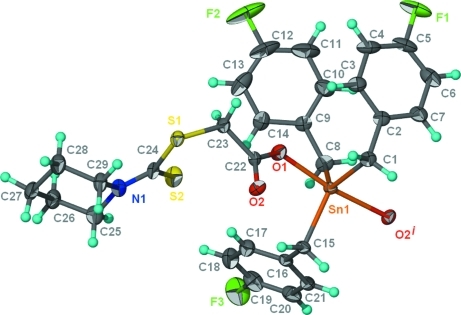
Anisotropic displacement ellipsoid plot (Barbour, 2001) of a portion of polymeric [Sn(C7H6F)3(C8H12NO2S2)]n; ellipsoids are drawn at the 70% probability level and H atoms are of arbitrary radius. The disorder is not shown. Symmetry code is given in Table 1.

## supplementary crystallographic information

### Comment

Trialkyltin carboxylates generally adopt linear carboxylate-bridged motifs; the repeat distance of such polymers is independent of the nature of the carboxylate group. This feature of trialkyltin carboxylates is also borne out in tris(4-fluorobenzyl)tin (*N*-pentamethylenecarbamothio)sulfanylacetate (Scheme I), whose repeat distance, *i.e.*, half the *b*-axis, of 5.23 Å, falls within the range of repeat distances reported (Ng *et al.*, 1989). The tin atom shows *trans*-C_3_SnO_2_ trigonal bipyramidal coordination; the tin atom is displaced by 0.113 (2) Å out of the C_3_Sn girdle in the direction of the covalently-bonded oxygen atom. The O-Sn-O angle is close to linearity (Table 1).

### Experimental

Tris(4-fluorobenzyl)tin hydroxide was prepared by hydrolysis of tris(4-fluorobenzyl)tin chloride in dilute sodium hydroxide. The hydroxide (1.0 g, 2.2 mmol) and *N*-piperinyldithiocarbamylacetic acid (0.48 g, 2.2 mmol) were heated in ethanol for an hour. The solution was filtered and then set aside for the growth of crystals.

### Refinement

Hydrogen atoms were placed in calculated positions (C–H 0.95–0.99 Å) and were included in the refinement in the riding model approximation, with *U*(H) set to 1.2*U*_eq_(C).

The carboxylate ion is disordered in the C–N(CH_2_)_5_ portion only; the sulfur atoms are ordered. Pairs of carbon-sulfur single-bond distances, carbon-sulfur double-bond distances, carbon_sulfur_-nitrogen distances, carbon_methylene_-nitrogen distances and carbon-carbon (methylene) distances were restrained to within 0.01 Å of each other. The displacement parameters of the minor (primed) atoms were restrained to be similar to those of the major (unprimed) atoms. The two C(S_2_)–N(C_2_) fragments were restrained to be within 0.01 Å of a plane. The pairs of non-bonded sulfur_single bond_-nitrogen and sulfur_double bond_-nitrogen distances were also restrained, though with a much tighter restraint.

The final difference Fourier map had a hole in the vicinity of O1.

### Crystal data

**Table:**

[Sn(C_7_H_6_F)_3_(C_8_H_12_NO_2_S_2_)]	*F*(000) = 1344
*M**_r_* = 664.35	*D*_x_ = 1.556 Mg m^−^^3^
Monoclinic, *P*2_1_/*n*	Mo *K*α radiation, λ = 0.71073 Å
Hall symbol: -P 2yn	Cell parameters from 9852 reflections
*a* = 11.8016 (7) Å	θ = 2.4–28.3°
*b* = 10.4572 (6) Å	µ = 1.10 mm^−^^1^
*c* = 23.0334 (13) Å	*T* = 100 K
β = 94.013 (1)°	Prism, colorless
*V* = 2835.6 (3) Å^3^	0.30 × 0.25 × 0.20 mm
*Z* = 4	

### Data collection

**Table:**

Bruker SMART APEX diffractometer	6498 independent reflections
Radiation source: fine-focus sealed tube	5328 reflections with *I* > 2σ(*I*)
graphite	*R*_int_ = 0.038
ω scans	θ_max_ = 27.5°, θ_min_ = 1.8°
Absorption correction: multi-scan (*SADABS*; Sheldrick, 1996)	*h* = −15→15
*T*_min_ = 0.735, *T*_max_ = 0.811	*k* = −13→13
25437 measured reflections	*l* = −29→29

### Refinement

**Table:**

Refinement on *F*^2^	Primary atom site location: structure-invariant direct methods
Least-squares matrix: full	Secondary atom site location: difference Fourier map
*R*[*F*^2^ > 2σ(*F*^2^)] = 0.034	Hydrogen site location: inferred from neighbouring sites
*wR*(*F*^2^) = 0.077	H-atom parameters constrained
*S* = 1.05	*w* = 1/[σ^2^(*F*_o_^2^) + (0.0254*P*)^2^ + 5.3214*P*] where *P* = (*F*_o_^2^ + 2*F*_c_^2^)/3
6498 reflections	(Δ/σ)_max_ = 0.001
371 parameters	Δρ_max_ = 0.50 e Å^−^^3^
17 restraints	Δρ_min_ = −1.19 e Å^−^^3^

### Fractional atomic coordinates and isotropic or equivalent isotropic displacement parameters (Å^2^)

**Table:**

	*x*	*y*	*z*	*U*_iso_*/*U*_eq_	Occ. (<1)
Sn1	0.710443 (15)	0.543605 (18)	0.737119 (9)	0.01476 (6)	
S1	0.57734 (6)	0.05444 (7)	0.62134 (3)	0.01883 (15)	
S2	0.68919 (7)	0.25659 (8)	0.55248 (4)	0.02778 (18)	
F1	0.20300 (17)	0.7560 (2)	0.79264 (11)	0.0494 (6)	
F2	0.2292 (2)	0.5457 (3)	0.56053 (13)	0.0778 (10)	
F3	1.0169 (2)	0.7386 (2)	0.52170 (10)	0.0508 (6)	
O1	0.61265 (18)	0.39691 (19)	0.68910 (10)	0.0219 (5)	
O2	0.71042 (17)	0.22277 (19)	0.71464 (9)	0.0199 (4)	
N1	0.7199 (2)	0.0057 (3)	0.54087 (13)	0.0219 (9)	0.631 (4)
N1'	0.6619 (3)	0.0131 (4)	0.52047 (19)	0.0219 (9)	0.37
C1	0.6237 (2)	0.5024 (3)	0.81369 (13)	0.0203 (6)	
H1A	0.6141	0.4088	0.8174	0.024*	
H1B	0.6699	0.5330	0.8485	0.024*	
C2	0.5100 (2)	0.5657 (3)	0.81084 (13)	0.0194 (6)	
C3	0.4159 (3)	0.5111 (3)	0.78054 (14)	0.0244 (7)	
H3	0.4231	0.4294	0.7632	0.029*	
C4	0.3121 (3)	0.5734 (4)	0.77516 (16)	0.0318 (8)	
H4	0.2479	0.5347	0.7551	0.038*	
C5	0.3044 (3)	0.6923 (4)	0.79952 (16)	0.0316 (8)	
C6	0.3936 (3)	0.7503 (3)	0.83053 (16)	0.0306 (8)	
H6	0.3853	0.8322	0.8475	0.037*	
C7	0.4965 (3)	0.6853 (3)	0.83628 (14)	0.0236 (7)	
H7	0.5590	0.7233	0.8580	0.028*	
C8	0.6459 (3)	0.6860 (3)	0.67581 (14)	0.0212 (6)	
H8A	0.6363	0.7671	0.6971	0.025*	
H8B	0.7037	0.7011	0.6474	0.025*	
C9	0.5355 (2)	0.6548 (3)	0.64269 (13)	0.0192 (6)	
C10	0.4343 (3)	0.7070 (4)	0.65835 (15)	0.0309 (8)	
H10	0.4356	0.7684	0.6888	0.037*	
C11	0.3307 (3)	0.6717 (5)	0.63062 (18)	0.0463 (11)	
H11	0.2617	0.7076	0.6420	0.056*	
C12	0.3303 (3)	0.5845 (5)	0.58683 (19)	0.0475 (11)	
C13	0.4278 (3)	0.5319 (4)	0.56833 (16)	0.0375 (9)	
H13	0.4253	0.4725	0.5370	0.045*	
C14	0.5303 (3)	0.5679 (3)	0.59670 (14)	0.0249 (7)	
H14	0.5988	0.5324	0.5845	0.030*	
C15	0.8782 (2)	0.4800 (3)	0.71871 (13)	0.0187 (6)	
H15A	0.9318	0.4967	0.7529	0.022*	
H15B	0.8769	0.3867	0.7114	0.022*	
C16	0.9180 (2)	0.5479 (3)	0.66665 (13)	0.0183 (6)	
C17	0.8872 (3)	0.5034 (3)	0.61081 (14)	0.0242 (7)	
H17	0.8426	0.4280	0.6062	0.029*	
C18	0.9200 (3)	0.5665 (3)	0.56162 (15)	0.0324 (8)	
H18	0.8983	0.5354	0.5237	0.039*	
C19	0.9847 (3)	0.6754 (3)	0.56939 (16)	0.0323 (8)	
C20	1.0173 (3)	0.7237 (3)	0.62352 (16)	0.0287 (7)	
H20	1.0617	0.7994	0.6277	0.034*	
C21	0.9836 (2)	0.6587 (3)	0.67181 (14)	0.0222 (6)	
H21	1.0058	0.6906	0.7095	0.027*	
C22	0.6302 (2)	0.2774 (3)	0.68712 (12)	0.0163 (6)	
C23	0.5386 (2)	0.2056 (3)	0.65075 (13)	0.0189 (6)	
H23A	0.5114	0.2615	0.6180	0.023*	
H23B	0.4738	0.1915	0.6751	0.023*	
C24	0.6704 (3)	0.1026 (4)	0.56691 (15)	0.0166 (13)	0.631 (4)
C25	0.7999 (3)	0.0258 (5)	0.49474 (17)	0.0273 (10)	0.631 (4)
H25A	0.8750	−0.0103	0.5077	0.033*	0.631 (4)
H25B	0.8097	0.1187	0.4884	0.033*	0.631 (4)
C26	0.7582 (5)	−0.0356 (5)	0.4389 (2)	0.0273 (11)	0.631 (4)
H26A	0.8159	−0.0255	0.4100	0.033*	0.631 (4)
H26B	0.6878	0.0076	0.4234	0.033*	0.631 (4)
C27	0.7341 (5)	−0.1788 (5)	0.4475 (2)	0.0275 (14)	0.631 (4)
H27A	0.6973	−0.2150	0.4112	0.033*	0.631 (4)
H27B	0.8064	−0.2249	0.4566	0.033*	0.631 (4)
C28	0.6562 (5)	−0.1965 (5)	0.4974 (2)	0.0292 (11)	0.631 (4)
H28A	0.5804	−0.1607	0.4857	0.035*	0.631 (4)
H28B	0.6470	−0.2889	0.5050	0.035*	0.631 (4)
C29	0.7039 (3)	−0.1307 (4)	0.55303 (17)	0.0224 (9)	0.631 (4)
H29A	0.6506	−0.1411	0.5840	0.027*	0.631 (4)
H29B	0.7774	−0.1699	0.5666	0.027*	0.631 (4)
C24'	0.6478 (3)	0.1059 (6)	0.5589 (3)	0.029 (3)	0.369 (4)
C25'	0.7185 (5)	0.0395 (8)	0.4665 (3)	0.0273 (10)	0.37
H25C	0.7472	0.1285	0.4672	0.033*	0.369 (4)
H25D	0.6632	0.0302	0.4324	0.033*	0.369 (4)
C26'	0.8152 (7)	−0.0511 (8)	0.4610 (4)	0.0273 (11)	0.37
H26C	0.8744	−0.0349	0.4927	0.033*	0.369 (4)
H26D	0.8491	−0.0365	0.4235	0.033*	0.369 (4)
C27'	0.7748 (9)	−0.1904 (9)	0.4642 (4)	0.0275 (14)	0.37
H27C	0.7178	−0.2082	0.4315	0.033*	0.369 (4)
H27D	0.8398	−0.2491	0.4609	0.033*	0.369 (4)
C28'	0.7223 (7)	−0.2116 (8)	0.5220 (4)	0.0292 (11)	0.37
H28C	0.6947	−0.3007	0.5244	0.035*	0.369 (4)
H28D	0.7801	−0.1972	0.5546	0.035*	0.369 (4)
C29'	0.6248 (5)	−0.1199 (6)	0.5267 (3)	0.0224 (9)	0.37
H29C	0.5644	−0.1395	0.4959	0.027*	0.369 (4)
H29D	0.5925	−0.1308	0.5649	0.027*	0.369 (4)

### Atomic displacement parameters (Å^2^)

**Table:**

	*U*^11^	*U*^22^	*U*^33^	*U*^12^	*U*^13^	*U*^23^
Sn1	0.01313 (9)	0.01198 (9)	0.01910 (10)	0.00144 (8)	0.00059 (7)	0.00057 (8)
S1	0.0223 (3)	0.0147 (3)	0.0196 (4)	−0.0040 (3)	0.0021 (3)	−0.0018 (3)
S2	0.0371 (4)	0.0163 (4)	0.0304 (5)	−0.0072 (3)	0.0054 (4)	0.0006 (3)
F1	0.0236 (10)	0.0570 (15)	0.0692 (16)	0.0201 (10)	0.0158 (10)	0.0259 (13)
F2	0.0422 (14)	0.109 (2)	0.076 (2)	−0.0369 (16)	−0.0362 (13)	0.0325 (18)
F3	0.0663 (16)	0.0503 (14)	0.0387 (13)	−0.0086 (12)	0.0230 (12)	0.0114 (11)
O1	0.0226 (11)	0.0145 (10)	0.0276 (12)	0.0009 (8)	−0.0042 (9)	−0.0019 (9)
O2	0.0204 (10)	0.0144 (10)	0.0238 (11)	−0.0009 (8)	−0.0049 (8)	0.0033 (9)
N1	0.029 (2)	0.0191 (16)	0.018 (2)	−0.0064 (18)	0.0059 (16)	−0.0010 (15)
N1'	0.029 (2)	0.0191 (16)	0.018 (2)	−0.0064 (18)	0.0059 (16)	−0.0010 (15)
C1	0.0197 (14)	0.0210 (14)	0.0204 (15)	−0.0020 (11)	0.0023 (12)	0.0036 (12)
C2	0.0194 (14)	0.0190 (15)	0.0200 (15)	−0.0025 (11)	0.0038 (11)	0.0052 (12)
C3	0.0199 (15)	0.0256 (16)	0.0275 (17)	−0.0024 (12)	0.0013 (13)	0.0032 (13)
C4	0.0148 (15)	0.046 (2)	0.034 (2)	−0.0019 (14)	0.0005 (13)	0.0118 (16)
C5	0.0184 (15)	0.038 (2)	0.040 (2)	0.0098 (14)	0.0113 (14)	0.0173 (16)
C6	0.0315 (18)	0.0217 (16)	0.041 (2)	0.0036 (13)	0.0173 (16)	0.0063 (15)
C7	0.0202 (15)	0.0235 (16)	0.0282 (17)	−0.0050 (12)	0.0091 (13)	0.0018 (13)
C8	0.0239 (15)	0.0146 (14)	0.0244 (16)	0.0028 (12)	−0.0032 (12)	0.0069 (12)
C9	0.0203 (14)	0.0169 (14)	0.0201 (15)	0.0011 (11)	−0.0004 (12)	0.0063 (12)
C10	0.0258 (16)	0.039 (2)	0.0282 (18)	0.0087 (15)	0.0018 (14)	0.0041 (15)
C11	0.0193 (17)	0.074 (3)	0.046 (2)	0.0072 (18)	−0.0001 (16)	0.018 (2)
C12	0.0280 (19)	0.068 (3)	0.044 (2)	−0.0182 (19)	−0.0180 (17)	0.022 (2)
C13	0.050 (2)	0.033 (2)	0.0270 (18)	−0.0134 (18)	−0.0155 (16)	0.0071 (16)
C14	0.0317 (17)	0.0224 (17)	0.0198 (16)	0.0013 (13)	−0.0029 (13)	0.0035 (12)
C15	0.0153 (13)	0.0154 (15)	0.0257 (16)	0.0013 (11)	0.0031 (11)	−0.0023 (12)
C16	0.0139 (12)	0.0161 (13)	0.0251 (15)	0.0020 (11)	0.0029 (11)	−0.0029 (12)
C17	0.0246 (15)	0.0193 (14)	0.0286 (17)	−0.0020 (12)	0.0016 (13)	−0.0034 (13)
C18	0.0383 (19)	0.034 (2)	0.0250 (18)	−0.0007 (15)	0.0039 (15)	−0.0038 (15)
C19	0.0365 (19)	0.0315 (19)	0.0306 (19)	0.0003 (15)	0.0145 (15)	0.0069 (15)
C20	0.0232 (16)	0.0233 (16)	0.041 (2)	−0.0021 (13)	0.0110 (14)	0.0004 (15)
C21	0.0175 (14)	0.0209 (15)	0.0284 (17)	−0.0005 (12)	0.0036 (12)	−0.0066 (13)
C22	0.0183 (13)	0.0146 (14)	0.0162 (14)	0.0004 (11)	0.0016 (11)	−0.0016 (11)
C23	0.0197 (14)	0.0130 (14)	0.0237 (16)	0.0012 (11)	−0.0007 (12)	−0.0037 (12)
C24	0.025 (3)	0.014 (3)	0.012 (3)	−0.007 (2)	0.004 (2)	−0.001 (2)
C25	0.031 (2)	0.026 (2)	0.026 (2)	−0.007 (2)	0.0096 (17)	−0.001 (2)
C26	0.033 (3)	0.033 (2)	0.016 (3)	−0.006 (3)	0.0051 (18)	−0.002 (2)
C27	0.029 (4)	0.032 (2)	0.022 (3)	−0.007 (3)	0.001 (2)	−0.009 (2)
C28	0.035 (3)	0.025 (2)	0.028 (3)	−0.011 (2)	0.010 (2)	−0.007 (2)
C29	0.033 (2)	0.018 (2)	0.017 (2)	−0.0037 (19)	0.0049 (16)	−0.0008 (17)
C24'	0.034 (6)	0.023 (7)	0.030 (7)	−0.003 (5)	−0.006 (5)	−0.001 (5)
C25'	0.031 (2)	0.026 (2)	0.026 (2)	−0.007 (2)	0.0096 (17)	−0.001 (2)
C26'	0.033 (3)	0.033 (2)	0.016 (3)	−0.006 (3)	0.0051 (18)	−0.002 (2)
C27'	0.029 (4)	0.032 (2)	0.022 (3)	−0.007 (3)	0.001 (2)	−0.009 (2)
C28'	0.035 (3)	0.025 (2)	0.028 (3)	−0.011 (2)	0.010 (2)	−0.007 (2)
C29'	0.033 (2)	0.018 (2)	0.017 (2)	−0.0037 (19)	0.0049 (16)	−0.0008 (17)

### Geometric parameters (Å, °)

**Table:**

Sn1—C1	2.144 (3)	C13—H13	0.9500
Sn1—C8	2.155 (3)	C14—H14	0.9500
Sn1—C15	2.158 (3)	C15—C16	1.498 (4)
Sn1—O1	2.175 (2)	C15—H15A	0.9900
Sn1—O2^i^	2.339 (2)	C15—H15B	0.9900
S1—C24'	1.793 (7)	C16—C17	1.392 (4)
S1—C23	1.792 (3)	C16—C21	1.394 (4)
S1—C24	1.796 (5)	C17—C18	1.390 (5)
S2—C24'	1.659 (7)	C17—H17	0.9500
S2—C24	1.662 (5)	C18—C19	1.376 (5)
F1—C5	1.369 (4)	C18—H18	0.9500
F2—C12	1.362 (4)	C19—C20	1.375 (5)
F3—C19	1.358 (4)	C20—C21	1.385 (5)
O1—C22	1.268 (3)	C20—H20	0.9500
O2—C22	1.241 (3)	C21—H21	0.9500
O2—Sn1^ii^	2.339 (2)	C22—C23	1.518 (4)
N1—C24	1.333 (6)	C23—H23A	0.9900
N1—C29	1.468 (5)	C23—H23B	0.9900
N1—C25	1.485 (5)	C25—C26	1.490 (6)
N1'—C24'	1.332 (8)	C25—H25A	0.9900
N1'—C29'	1.468 (7)	C25—H25B	0.9900
N1'—C25'	1.479 (7)	C26—C27	1.540 (6)
C1—C2	1.494 (4)	C26—H26A	0.9900
C1—H1A	0.9900	C26—H26B	0.9900
C1—H1B	0.9900	C27—C28	1.530 (6)
C2—C3	1.392 (4)	C27—H27A	0.9900
C2—C7	1.395 (4)	C27—H27B	0.9900
C3—C4	1.386 (4)	C28—C29	1.527 (6)
C3—H3	0.9500	C28—H28A	0.9900
C4—C5	1.369 (5)	C28—H28B	0.9900
C4—H4	0.9500	C29—H29A	0.9900
C5—C6	1.371 (5)	C29—H29B	0.9900
C6—C7	1.389 (4)	C25'—C26'	1.495 (8)
C6—H6	0.9500	C25'—H25C	0.9900
C7—H7	0.9500	C25'—H25D	0.9900
C8—C9	1.499 (4)	C26'—C27'	1.535 (9)
C8—H8A	0.9900	C26'—H26C	0.9900
C8—H8B	0.9900	C26'—H26D	0.9900
C9—C10	1.384 (4)	C27'—C28'	1.525 (8)
C9—C14	1.394 (4)	C27'—H27C	0.9900
C10—C11	1.389 (5)	C27'—H27D	0.9900
C10—H10	0.9500	C28'—C29'	1.507 (8)
C11—C12	1.359 (6)	C28'—H28C	0.9900
C11—H11	0.9500	C28'—H28D	0.9900
C12—C13	1.370 (6)	C29'—H29C	0.9900
C13—C14	1.386 (5)	C29'—H29D	0.9900
			
C1—Sn1—C8	120.72 (12)	F3—C19—C18	118.8 (3)
C1—Sn1—C15	126.65 (11)	C20—C19—C18	122.6 (3)
C8—Sn1—C15	111.80 (12)	C19—C20—C21	118.1 (3)
C1—Sn1—O1	90.56 (10)	C19—C20—H20	121.0
C8—Sn1—O1	90.20 (10)	C21—C20—H20	121.0
C15—Sn1—O1	98.28 (10)	C20—C21—C16	121.9 (3)
C1—Sn1—O2^i^	88.16 (10)	C20—C21—H21	119.1
C8—Sn1—O2^i^	82.46 (10)	C16—C21—H21	119.1
C15—Sn1—O2^i^	89.94 (9)	O2—C22—O1	123.7 (3)
O1—Sn1—O2^i^	170.59 (7)	O2—C22—C23	122.8 (3)
C24'—S1—C23	100.5 (2)	O1—C22—C23	113.3 (2)
C23—S1—C24	101.60 (18)	C22—C23—S1	117.0 (2)
C22—O1—Sn1	129.16 (19)	C22—C23—H23A	108.0
C22—O2—Sn1^ii^	151.17 (19)	S1—C23—H23A	108.0
C24—N1—C29	125.9 (4)	C22—C23—H23B	108.0
C24—N1—C25	122.3 (4)	S1—C23—H23B	108.0
C29—N1—C25	111.8 (4)	H23A—C23—H23B	107.3
C24'—N1'—C29'	125.0 (5)	N1—C24—S2	125.3 (3)
C24'—N1'—C25'	120.6 (6)	N1—C24—S1	114.2 (3)
C29'—N1'—C25'	114.5 (5)	S2—C24—S1	120.5 (3)
C2—C1—Sn1	110.7 (2)	N1—C25—C26	111.6 (3)
C2—C1—H1A	109.5	N1—C25—H25A	109.3
Sn1—C1—H1A	109.5	C26—C25—H25A	109.3
C2—C1—H1B	109.5	N1—C25—H25B	109.3
Sn1—C1—H1B	109.5	C26—C25—H25B	109.3
H1A—C1—H1B	108.1	H25A—C25—H25B	108.0
C3—C2—C7	117.9 (3)	C25—C26—C27	111.1 (4)
C3—C2—C1	121.5 (3)	C25—C26—H26A	109.4
C7—C2—C1	120.5 (3)	C27—C26—H26A	109.4
C4—C3—C2	121.4 (3)	C25—C26—H26B	109.4
C4—C3—H3	119.3	C27—C26—H26B	109.4
C2—C3—H3	119.3	H26A—C26—H26B	108.0
C5—C4—C3	118.3 (3)	C28—C27—C26	109.9 (4)
C5—C4—H4	120.9	C28—C27—H27A	109.7
C3—C4—H4	120.9	C26—C27—H27A	109.7
C4—C5—F1	118.5 (3)	C28—C27—H27B	109.7
C4—C5—C6	123.0 (3)	C26—C27—H27B	109.7
F1—C5—C6	118.5 (3)	H27A—C27—H27B	108.2
C5—C6—C7	117.8 (3)	C29—C28—C27	111.7 (4)
C5—C6—H6	121.1	C29—C28—H28A	109.3
C7—C6—H6	121.1	C27—C28—H28A	109.3
C6—C7—C2	121.5 (3)	C29—C28—H28B	109.3
C6—C7—H7	119.2	C27—C28—H28B	109.3
C2—C7—H7	119.2	H28A—C28—H28B	107.9
C9—C8—Sn1	116.0 (2)	N1—C29—C28	108.8 (3)
C9—C8—H8A	108.3	N1—C29—H29A	109.9
Sn1—C8—H8A	108.3	C28—C29—H29A	109.9
C9—C8—H8B	108.3	N1—C29—H29B	109.9
Sn1—C8—H8B	108.3	C28—C29—H29B	109.9
H8A—C8—H8B	107.4	H29A—C29—H29B	108.3
C10—C9—C14	117.6 (3)	N1'—C24'—S2	125.5 (5)
C10—C9—C8	121.1 (3)	N1'—C24'—S1	113.6 (5)
C14—C9—C8	121.2 (3)	S2—C24'—S1	120.9 (4)
C9—C10—C11	121.5 (4)	N1'—C25'—C26'	110.2 (5)
C9—C10—H10	119.2	N1'—C25'—H25C	109.6
C11—C10—H10	119.2	C26'—C25'—H25C	109.6
C12—C11—C10	118.5 (4)	N1'—C25'—H25D	109.6
C12—C11—H11	120.8	C26'—C25'—H25D	109.6
C10—C11—H11	120.8	H25C—C25'—H25D	108.1
C11—C12—F2	119.1 (4)	C25'—C26'—C27'	110.9 (8)
C11—C12—C13	122.8 (3)	C25'—C26'—H26C	109.5
F2—C12—C13	118.1 (4)	C27'—C26'—H26C	109.5
C12—C13—C14	117.9 (4)	C25'—C26'—H26D	109.5
C12—C13—H13	121.0	C27'—C26'—H26D	109.5
C14—C13—H13	121.0	H26C—C26'—H26D	108.1
C13—C14—C9	121.7 (3)	C28'—C27'—C26'	109.0 (8)
C13—C14—H14	119.2	C28'—C27'—H27C	109.9
C9—C14—H14	119.2	C26'—C27'—H27C	109.9
C16—C15—Sn1	110.79 (19)	C28'—C27'—H27D	109.9
C16—C15—H15A	109.5	C26'—C27'—H27D	109.9
Sn1—C15—H15A	109.5	H27C—C27'—H27D	108.3
C16—C15—H15B	109.5	C29'—C28'—C27'	109.2 (8)
Sn1—C15—H15B	109.5	C29'—C28'—H28C	109.8
H15A—C15—H15B	108.1	C27'—C28'—H28C	109.8
C17—C16—C21	117.7 (3)	C29'—C28'—H28D	109.8
C17—C16—C15	120.3 (3)	C27'—C28'—H28D	109.8
C21—C16—C15	122.0 (3)	H28C—C28'—H28D	108.3
C18—C17—C16	121.7 (3)	N1'—C29'—C28'	111.2 (5)
C18—C17—H17	119.2	N1'—C29'—H29C	109.4
C16—C17—H17	119.2	C28'—C29'—H29C	109.4
C19—C18—C17	118.1 (3)	N1'—C29'—H29D	109.4
C19—C18—H18	121.0	C28'—C29'—H29D	109.4
C17—C18—H18	121.0	H29C—C29'—H29D	108.0
F3—C19—C20	118.6 (3)		
			
C1—Sn1—O1—C22	−83.6 (3)	C19—C20—C21—C16	−0.3 (5)
C8—Sn1—O1—C22	155.7 (3)	C17—C16—C21—C20	0.2 (4)
C15—Sn1—O1—C22	43.6 (3)	C15—C16—C21—C20	−177.9 (3)
C8—Sn1—C1—C2	10.2 (3)	Sn1^ii^—O2—C22—O1	149.7 (3)
C15—Sn1—C1—C2	178.85 (19)	Sn1^ii^—O2—C22—C23	−26.1 (6)
O1—Sn1—C1—C2	−80.4 (2)	Sn1—O1—C22—O2	0.8 (4)
O2^i^—Sn1—C1—C2	90.3 (2)	Sn1—O1—C22—C23	177.03 (18)
Sn1—C1—C2—C3	82.4 (3)	O2—C22—C23—S1	−27.3 (4)
Sn1—C1—C2—C7	−94.3 (3)	O1—C22—C23—S1	156.4 (2)
C7—C2—C3—C4	0.6 (5)	C24'—S1—C23—C22	−80.7 (2)
C1—C2—C3—C4	−176.3 (3)	C24—S1—C23—C22	−70.5 (2)
C2—C3—C4—C5	1.3 (5)	C29—N1—C24—S2	−179.80 (10)
C3—C4—C5—F1	177.8 (3)	C25—N1—C24—S2	0.15 (17)
C3—C4—C5—C6	−2.2 (5)	C29—N1—C24—S1	0.16 (15)
C4—C5—C6—C7	1.2 (5)	C25—N1—C24—S1	−179.89 (8)
F1—C5—C6—C7	−178.9 (3)	C24'—S2—C24—N1	100.1 (16)
C5—C6—C7—C2	0.9 (5)	C24'—S2—C24—S1	−79.9 (16)
C3—C2—C7—C6	−1.7 (4)	C24'—S1—C24—N1	−100.3 (15)
C1—C2—C7—C6	175.2 (3)	C23—S1—C24—N1	174.76 (15)
C1—Sn1—C8—C9	−67.4 (3)	C24'—S1—C24—S2	79.7 (15)
C15—Sn1—C8—C9	122.4 (2)	C23—S1—C24—S2	−5.27 (16)
O1—Sn1—C8—C9	23.4 (2)	C24—N1—C25—C26	−119.8 (4)
O2^i^—Sn1—C8—C9	−150.7 (2)	C29—N1—C25—C26	60.2 (4)
Sn1—C8—C9—C10	101.0 (3)	N1—C25—C26—C27	−55.1 (6)
Sn1—C8—C9—C14	−76.1 (3)	C25—C26—C27—C28	52.1 (6)
C14—C9—C10—C11	1.9 (5)	C26—C27—C28—C29	−53.8 (6)
C8—C9—C10—C11	−175.3 (3)	C24—N1—C29—C28	120.1 (4)
C9—C10—C11—C12	−0.6 (6)	C25—N1—C29—C28	−59.9 (3)
C10—C11—C12—F2	177.8 (4)	C27—C28—C29—N1	57.6 (5)
C10—C11—C12—C13	−1.1 (6)	C29'—N1'—C24'—S2	−179.98 (10)
C11—C12—C13—C14	1.4 (6)	C25'—N1'—C24'—S2	0.02 (17)
F2—C12—C13—C14	−177.6 (3)	C29'—N1'—C24'—S1	0.01 (15)
C12—C13—C14—C9	0.0 (5)	C25'—N1'—C24'—S1	−179.99 (8)
C10—C9—C14—C13	−1.6 (5)	C24—S2—C24'—N1'	−98.2 (16)
C8—C9—C14—C13	175.6 (3)	C24—S2—C24'—S1	81.8 (16)
C1—Sn1—C15—C16	−169.27 (19)	C23—S1—C24'—N1'	−164.49 (17)
C8—Sn1—C15—C16	0.3 (2)	C24—S1—C24'—N1'	98.5 (15)
O1—Sn1—C15—C16	93.8 (2)	C23—S1—C24'—S2	15.51 (17)
O2^i^—Sn1—C15—C16	−81.6 (2)	C24—S1—C24'—S2	−81.5 (15)
Sn1—C15—C16—C17	−84.4 (3)	C24'—N1'—C25'—C26'	125.9 (6)
Sn1—C15—C16—C21	93.6 (3)	C29'—N1'—C25'—C26'	−54.1 (6)
C21—C16—C17—C18	−0.1 (5)	N1'—C25'—C26'—C27'	55.3 (9)
C15—C16—C17—C18	178.0 (3)	C25'—C26'—C27'—C28'	−58.9 (10)
C16—C17—C18—C19	0.1 (5)	C26'—C27'—C28'—C29'	58.6 (10)
C17—C18—C19—F3	−179.7 (3)	C24'—N1'—C29'—C28'	−124.7 (6)
C17—C18—C19—C20	−0.3 (5)	C25'—N1'—C29'—C28'	55.3 (6)
F3—C19—C20—C21	179.8 (3)	C27'—C28'—C29'—N1'	−56.7 (9)
C18—C19—C20—C21	0.4 (5)		

Symmetry codes: (i) −*x*+3/2, *y*+1/2, −*z*+3/2; (ii) −*x*+3/2, *y*−1/2, −*z*+3/2.
